# Assessing the molecular structure basis for biomass recalcitrance during dilute acid and hydrothermal pretreatments

**DOI:** 10.1186/1754-6834-6-15

**Published:** 2013-01-28

**Authors:** Yunqiao Pu, Fan Hu, Fang Huang, Brian H Davison, Arthur J Ragauskas

**Affiliations:** 1 Institute of Paper Science and Technology, Georgia Institute of Technology, Atlanta, GA, USA; 2 BioEnergy Science Center, School of Chemistry and Biochemistry, Georgia Institute of Technology, Atlanta, GA, USA; 3 Biosciences Division, Oak Ridge National Laboratory, Oak Ridge, TN, USA; 4 BioEnergy Science Center, Oak Ridge, TN, USA

**Keywords:** Biomass recalcitrance, Dilute acid pretreatment, Hydrothermal pretreatment, Cellulose structure, Structural transformation

## Abstract

The production of cellulosic ethanol from biomass is considered a promising alternative to reliance on diminishing supplies of fossil fuels, providing a sustainable option for fuels production in an environmentally compatible manner. The conversion of lignocellulosic biomass to biofuels through a biological route usually suffers from the intrinsic recalcitrance of biomass owing to the complicated structure of plant cell walls. Currently, a pretreatment step that can effectively reduce biomass recalcitrance is generally required to make the polysaccharide fractions locked in the intricacy of plant cell walls to become more accessible and amenable to enzymatic hydrolysis. Dilute acid and hydrothermal pretreatments are attractive and among the most promising pretreatment technologies that enhance sugar release performance. This review highlights our recent understanding on molecular structure basis for recalcitrance, with emphasis on structural transformation of major biomass biopolymers (i.e., cellulose, hemicellulose, and lignin) related to the reduction of recalcitrance during dilute acid and hydrothermal pretreatments. The effects of these two pretreatments on biomass porosity as well as its contribution on reduced recalcitrance are also discussed.

## Introduction

With the increasing concerns on diminishing fossil fuel resources, climate change and energy security, the utilization of renewable and sustainable resources for the production of fuels, chemicals and materials has become a global research theme and in the future will play an important role in our energy portfolio. Among them, biofuels produced from biomass have taken a lead position as a viable option to petroleum-derived fuels. The production of cellulosic ethanol through biological route has garnered extensive interest over the past decade with one of its major advantages being that it is based on non-food lignocellulosics [1,2]. This route is contingent on the efficient hydrolysis of plant polysaccharides to monosaccharides and usually involves three steps: pretreatment, enzymatic hydrolysis, and fermentation. Currently, one of the key challenges for this route is the development of efficient and cost-competitive pretreatment technologies that can reduce biomass recalcitrance thus enabling better sugar release performance through enzymatic hydrolysis [3-6].

Lignocellulosic biomass consists of three major structural biopolymers, namely cellulose, hemicellulose, and lignin, with each of these components having a unique and complex structure. Cellulose is a linear chain homopolymer consisting of (1→4)-β-D-glucopyranosyl units with a varying degree of polymerization (DP) up to ~10,000. The cellulose chain has a tendency to form intra- and inter-molecular hydrogen bonds through hydroxyl groups on its glucose units, which promotes cellulose aggregations and lead to a supramolecular structure with crystalline and amorphous domains. On the other hand, hemicellulose consists of a broad class of mixed heteroglycans of pentoses and hexanoses (mainly xylose and mannose) which link together and frequently have branching and substitution groups. Lignin is an irregular polyphenolic biopolymer constructed of phenylpropanoid monomers with various degrees of methoxylation that are biosynthesized into a complex and highly heterogeneous aromatic macromolecule. According to our current understanding, the plant cell wall microstructure is a lignin and polysaccharides matrix in which these biopolymers are intimately associated with each other [7,8]. In addition, plant cell walls generally are composed of three layers of anatomical regions (i.e., the middle lamella, the primary wall, and the secondary wall), with the thickness of each layer and its constituents composition varying in different cell types, tissues and plant species. The structural complexity of plant cell walls causes plant biomass to be resistant to enzymatic and microbial deconstruction, which is defined as biomass recalcitrance [9].

To date, pretreatment is generally required as the first step for the biological conversion of lignocellulosic biomass to biofuels. The purpose of pretreatment is to reduce biomass recalcitrance by altering cell wall structural features so that the polysaccharide fractions (mainly cellulose) locked in the intricacy of plant cell walls can become more accessible and amenable to enzymatic hydrolysis [3,6,10]. Numerous pretreatment approaches including physical, chemical, and physic-chemical and biological techniques have been tried/developed to reduce recalcitrance and improve sugar yields of cellulosic biomass. Dependent on the pretreatment parameters, several key properties of biomass are altered and believed to impact the recalcitrance of pretreated biomass including the resulting biomass constituents, cellulose crystallinity and ultrastructure, lignin/hemicellulose structures, cellulose degree of polymerization, and accessibility (i.e., pore size and pore volume).

Dilute acid (DA) pretreatment has been considered to be among the leading and most promising pretreatment technologies that can enhance biomass sugar release performance [3,6,11]. DA pretreatment involves the treatment of biomass with a combination of an acidic pH, heat and pressure with residence times ranging from less than a minute to 1 h, which is generally carried out using 0.4 – 2.0% (w/w) H_2_SO_4_ at a temperature of 140 - 200°C. Hydrothermal pretreatment, also called autohydrolysis or hot water pretreatment, is another attractive pretreatment process as it uses only water as a reaction medium without additional chemicals and lower cost of construction materials can be used. Hydrothermal pretreatment is usually carried out at relatively high temperature (140-220°C) under mild acidic conditions which come about largely from the release of organic acids from biomass components and a decrease in the pK_w_ of water at the elevated temperature. These acidic pretreatment processes are effective in producing high sugar yields from a wide range of lignocellulosic biomass. DA and hydrothermal pretreatments cause structural changes of lignin and cellulose as well as solubilization of hemicellulose, which in turn contribute to the reduction of biomass recalcitrance. This review highlights recent developments in assessing the molecular basis of recalcitrance, with focus on lignin, cellulose and hemicellulose structural transformations related to reducing recalcitrance during dilute acid and hydrothermal pretreatments. The effects of dilute acid and hydrothermal pretreatments on biomass porosity are also discussed.

## Lignin structural alterations and recalcitrance

**Figure 1:**
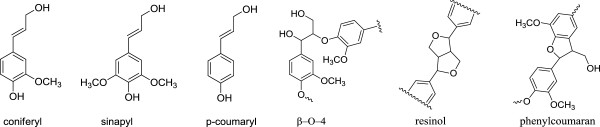
Typical phenylpropanoid precursors employed in the biosynthesis of lignin in plant biomass and some primary interunit linkages in lignin macromolecules.

Lignin is a polyphenolic polymer that accounts for ~ 15-35% of plant biomass. Three types of phenylpropanoid units are generally considered as major precursors for biosynthesis of lignin: coniferyl, sinapyl, and *p*-coumaryl alcohol (see Figure 1), which give rise to guaiacyl (G), syringyl (S) and *p*-hydroxyphenyl (H) units respectively in its structure [12]. Generally, lignin in softwoods is mainly composed of guaiacyl units with small amounts of *p*-hydroxyphenyl units existed, while lignin in hardwoods primarily consists of both guaiacyl and syringyl units including a minor amount of *p*-hydroxyphenyl units. Lignin in grasses typically contains all the three types of monolignol units, with peripheral groups (i.e., hydroxycinnamic acids) incorporating into its core structure [8,12]. The lignin macromolecule is primarily connected via carbon-carbon and carbon-oxygen (see Figure 1) bonds among its phenylpropanoid building blocks with aryl ether bonds (β-O-4) being the most common and important interunit linkage.

Lignin is considered the most recalcitrant component of the major plant cell wall biopolymers. It is found primarily in the secondary cell wall and plays a major role in pathogen resistance, water regulation, and conferring strength for the integrity of the cell wall structure. The effects of lignin on biomass enzymatic digestibility have received extensive attention. In general, it is perceived that the lower lignin content a plant biomass has, the higher the bioavailability of the substrate for bioethanol generation. However, a recent study by Studer et al. [13] has identified several unusual *Populus* that did not follow the dependency of sugar release performance on lignin content. Along with lignin content, other prominent lignin related factors that impact biomass digestibility may include lignin composition, its chemical structures, and lignin-carbohydrate complex (LCC) linkages presented in biomass.

### Lignin removal and pseudo-lignin formation

It is commonly assumed that the presence of lignin in biomass restricts enzymatic hydrolysis primarily by physically impeding the accessibility of cellulase to cellulose and unproductively binding cellulase. DA and hydrothermal pretreatments can cause fragmentation of lignin, usually resulting in a slight delignification (i.e., lignin removal) in biomass depending on the pretreatment severity [14-16]. For example, Silverstein et al. [15] reported a lignin reduction of ~2-24% in cotton stalk dilute acid pretreatment. Likewise, Liu and Wyman [16] observed less than 12% lignin removal in hot water pretreatment of corn stover after 20 min at 200°C. The lignin removal during dilute acid and hydrothermal pretreatment was shown to contribute to the improved cellulose digestibility [17-19]. High-resolution measurement of the microfibrillar nanoscale architecture of cell walls by Ding et al. [20] demonstrates that cellulose digestion is primarily facilitated by enabling enzyme access to the hydrophobic cellulose face and the data suggests that ideal pretreatments should maximize lignin removal and minimize polysaccharide modification/degradation, thereby retaining the essentially native microfibrillar structure. While Ishizawa et al. [17] observed that partial delignification of corn stover during dilute acid pretreatment improved cellulose digestibility, they also reported that near complete lignin removal (lignin content below 5%) in the corn stover after dilute acid pretreatment reduced cellulose conversion and particularly this effect was found to be enhanced in samples with lower xylan contents (< 4%). This effect was proposed to be attributed to decreased cellulase accessibility due to aggregation of adjacent cellulose microfibrils that was caused by elimination of the lignin spacer. These results suggest that there could be a balance between lignin removal and a need to retain some lignin and remain cell wall architecture with minimum alteration/degradation of polysaccharides to provide an optimal pretreated biomass for subsequent enzymatic deconstruction. On the other hand, some recent data suggests that lignin removal does not significantly contribute to the reduction of recalcitrance during DA and hydrothermal pretreatment. DeMartini et al. [21] investigated the cell wall compositional changes in *Populus* biomass during hydrothermal pretreatment of different times at 180°C and demonstrated that glucose yield from enzymatic hydrolysis improved even though lignin removal during hydrothermal pretreatment was minimal. The authors suggested that lignin content per se does not affect recalcitrance significantly; rather, the integration of lignin and polysaccharides within the cell wall, and their associations with one another and with other wall components, play a larger role that contributes to biomass recalcitrance.

**Figure 2:**
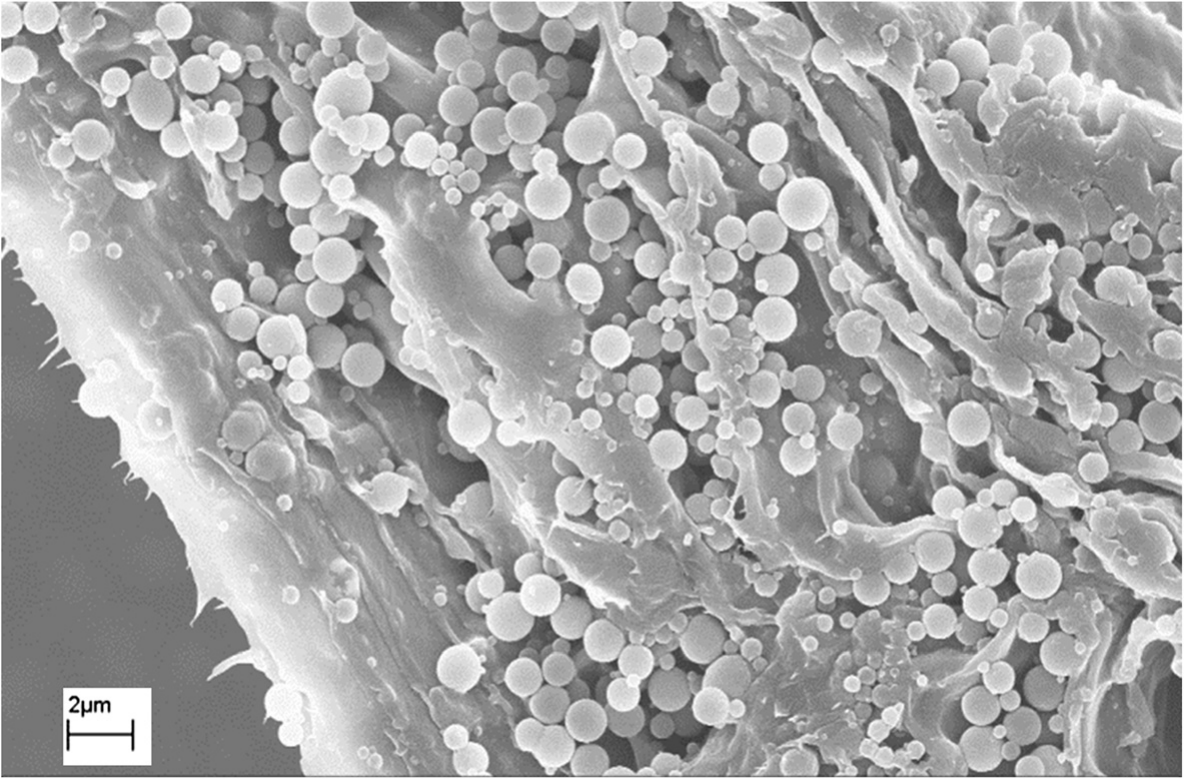
SEM image of pseudo-lignin deposition on surface of poplar holocellulose after dilute acid pretreatment.

DA and hydrothermal pretreatments generally lead to an insignificant delignification, thus the lignin content in the pretreated biomass can be comparable to or higher than that in the starting material [13,22,23]. For example, a recent study by Cao et al. [24] reported lignin contents (~ 24.4-25.9%) in the pretreated poplar similar to the unpretreated control (24.6%) after dilute acid pretreatment at 170°C over the range of 0.3-26.8 min. A ~ 2-6% lignin content increase was observed in pretreated poplar after dilute acid pretreatment at 140 – 180°C [13]. Similarly, Samuel et al. [23] documented a 10% increase in lignin content in pretreated switchgrass after DA pretreatment at 190°C with the residence time of 1 min. The relatively comparable/higher lignin content observed in pretreated biomass can be mostly attributed to the concomitant loss of polysaccharides and/or pseudo-lignin formation during DA and hydrothermal pretreatment. Sannigrahi et al. reported that acid catalyzed dehydration of carbohydrates during DA pretreatment was responsible for the formation of pseudo-lignin [25]. The formed pseudo-lignin usually has spherical structures and deposits on cell surfaces in pretreated biomass after dilute acid pretreatment (see Figure 2) [25,26]. Hu et al. studied the impacts of pseudo-lignin on cellulose enzymatic hydrolysis and observed a lower sugar yield with increased pseudo-lignin content and this inhibition effect was shown to be more significant than lignin [26]. In addition, lignin or its fragments were also reported to migrate to biomass surface during DA pretreatment where they deposited as a lignin droplets or balls [27-29]. Using SEM and TEM imaging techniques, Donohoe et al. [21] revealed that dilute acid pretreatment above the melting temperature of lignin caused lignin to coalesce into larger molten bodies that migrate within and out of the cell wall, and then re-deposit as droplets on the surface of biomass cell walls. Similar to pseudo-lignin, the re-deposited lignin droplets on the biomass surface were observed to have detrimental impacts on the enzymatic hydrolysis [27], which was attributed to its limiting enzyme access to cellulose as physical barrier and tending to irreversibly bind to enzymes although the exact mechanism of cellulase-lignin interaction is unclear. It should be also noted that the nonproductive binding of hydrolytic enzymes to lignin as a significant or insignificant factor that affects enzymatic hydrolysis performance is still highly debated. Recently, Li et al. [30] investigated the mechanisms of cellulase inhibition caused by lignin droplets on Avicel cellulose and proposed that a “traffic jam” effect on the Avicel surface produced by lignin droplets played a major role in cellulase inhibition and nonspecific binding was not the key source of inhibition especially at high enzyme loadings. Apparently, pseudo-lignin formation and re-deposited lignin droplets in the pretreated substrates are not desired due to their inhibitions on enzymatic hydrolysis. On the other hand, Donohoe et al. [21] argued that the process of lignin migrating/re-locating to a more localized, concentrated distribution would likely increase the accessibility of individual cellulose microfibrils deep within the cell wall. The authors suggest that the re-localization of lignin during DA and hydrothermal pretreatment is likely to be as important as lignin removal to improve digestibility as the pattern of lignin re-localization can dramatically open up the structure of the cell wall matrix and improve the accessibility of the majority of cellulose microfibrils, which likely explains a critical mechanism for the enhanced digestibility of DA and hydrothermal pretreated biomass.

### Aryl ether linkages cleavage

Under acidic pretreatment conditions, the predominant reactions in lignin are fragmentation by acidolysis of aryl ether linkages (primarily β-O-4 linkages) and acid catalyzed recondensation [31-33], while linkages such as resinol and phenylcoumaran subunits (see Figure 1) are fairly stable [24,34]. The β-O-4 linkages in lignin are susceptible to acidic hydrolysis and the pretreatments generally result in its lower relative content in the pretreated biomass [23,31]. For example, Samuel et al. [23] demonstrated that dilute acid pretreatment led to a 36% decrease of β-O-4 linkages in lignin of pretreated switchgrass. Change in monolignol S/G ratio in lignin was another prominent structural alteration observed after dilute acid pretreatment. Cao et al. [24] proposed that syringyl units (i.e., etherified) were more readily removed as a result of β-O-4 linkage cleavage, thus leading to a lower proportion of total S units remaining in pretreated poplar. Recently, Jung et al. [35] investigated the surface of poplar after dilute acid pretreatment and observed the intensity of S-lignin dramatically decreased while the content of G-lignin units doubled on the surface of poplar stem. Comparing free phenolic OH groups that associate with S and G lignin using ^31^P-NMR, Moxley et al. [36] demonstrated that lignin in DA pretreated biomass had a greater increase in content of phenolic S units than phenolic G units, mostly due to the liable cleavage of β-O-4 linkages in lignin syringyl units.

Compared to dilute acid pretreatment, hydrothermal pretreatment has milder acidic conditions as the hydrolysis is catalyzed by the organic acids released from biomass components during the process. From a chemistry point of view, the types of reaction occurring to lignin during hydrothermal pretreatment are similar to those taking place in dilute acid pretreatment although frequently to a lesser extent. Thus hydrothermal pretreatment was also found to lead to a decrease in β-O-4 linkages in lignin (i.e., acidolysis of β-O-4 linkages). Leschinsky et al. [34] revealed that the S/G ratio in *E. globulus* and poplar wood remained relatively constant during autohydrolysis, suggesting no preferential hydrolysis and/or condensation of S or G units occurred under the conditions employed. Both DA and hydrothermal pretreatments were reported to result in an increase of phenolic OH groups in lignin apparently resulting from cleavage of aryl ether linkages [24,36].

The cleavage of aryl ether linkage in lignin during DA and hydrothermal pretreatment can result in lignin fragmentation thus disrupting the biomass cell wall matrix and facilitating cellulase accessibility to cellulose. In addition, the acidic pretreatment might also cause cleavage of some labile linkages between lignin and carbohydrates (mainly hemicellulose) therefore facilitating hemicellulose dissolution, which in turn increases pore volume and available surface area in pretreated biomass. Recently, using glycome profiling DeMartini et al. [21] observed the disruptiveness of lignin’s association with pectins, arabinogalactans, and some xylans even by a relatively mild hydrothermal pretreatment of poplar biomass (180°C, 11 min). The disruption of lignin-arabinogalactan/pectin/xylan associations together with other changes such as the loss of arabinogalactan/xylan occurring in the cell wall was suggested to contribute to a reduction in recalcitrance resulting in an increase in digestibility of pretreated poplar biomass. While lignin-carbohydrate complex (LCC) linkages have been long considered among the factors contributing biomass recalcitrance, the role it plays to reducing recalcitrance and the cleavage rates of these linkages during DA and hydrothermal pretreatment is still not fully understood. Determining the susceptibility of various LCC linkages during DA and hydrothermal pretreatment as well as the mechanisms and kinetics for these reactions will provide critical information for the development of optimal pretreatment strategies to reduce cell wall recalcitrance.

### Lignin molecular weights

Changes in molecular weights of lignin can provide important insights into lignin’s fragmentation and recondensation reactions during dilute acid and hydrothermal pretreatment. While cleavage of β-O-4 linkages can result in a decrease in molecular weight of lignin, condensation reactions usually lead to a condensed and heterogeneous lignin structure with an increase in molecular size. Samuel et al. [23] observed a ~20% lower number-average molecular weight in lignin in the dilute acid pretreated switchgrass at 190°C, which was attributed to the short pretreatment time (residence time of 1 min) and limited opportunities for recondensation. Similarly, Cao et al. [24] reported that the molecular weight of lignin in poplar showed a small initial decrease of ~ 12% at a short dilute acid pretreatment time (0.3 min), which was probably due to the dominance of aryl ether linkage cleavage at the early pretreatment stage. As the pretreatment time extended, recondensation reactions became dominant, resulting in an increased molecular weight. Hydrothermal pretreatment usually results in a decrease of molecular weight in biomass lignin, most likely due to fragmentation dominating over condensation under the mild acidic conditions. For example, Leschinsky et al. [34] reported that autohydrolysis of *E. globulus* wood at 170°C led to a molecular weight loss in lignin. Similarly, when poplar was subjected to autohydrolysis pretreatment at 180°C, a reduction of lignin molecular weight was also observed [37]. On the other hand, hydrothermal pretreatment with high severity could also result in an increased molecular weight in lignin [31]. These results suggest that lignin molecular weight in the pretreated substrates appears to depend on the competition between fragmentation and condensation which is contingent on the pretreatment conditions and severity.

Recently, Ziebell et al. [38] reported that lignin in transgenic alfalfa with lower molecular weight had better extractability during chemical processing. During dilute acid and hydrothermal pretreatment, a decrease in molecular weight of native lignin would facilitate its dissolution and/or migration to the surface in the reaction media. Thus care needs to be taken to avoid lignin recondensation becoming dominant during DA and hydrothermal pretreatments, as well as to increase delignification and reduce lignin droplets deposition.

### Wild type and transgenic biomass

A previous report by Davison et al. [39] has identified lignin content and S/G ratio in *Populus* as dominant factors affecting xylose release upon dilute sulfuric acid hydrolysis. Lignin S/G ratios have been considered among the major structural features that impact the recalcitrance of plant biomass [13,39]. Recently, Studer et al. [13] examined the influence of lignin content and S/G ratio in natural *Populus* variants covering a wide range of lignin content (15.7-27.9%) and S/G ratio (1.0-3.0) on sugar release performance. They observed that total sugar release for dilute acid pretreated poplar had a strong negative correlation with lignin content only for pretreated samples with an S/G ratio < 2.0; with S/G ratio > 2.0, the negative influence of lignin was less pronounced. Poplar species with higher S/G ratios generally had a higher sugar release yield from enzymatic hydrolysis after dilute acid pretreatment; however, for substrates without pretreatment, sugar release was observed to increase when lignin content was below 20%, irrespective of the S/G ratio. Furthermore, certain samples with average lignin content and S/G ratios exhibited exceptional sugar release, suggesting that factors beyond lignin content and S/G ratio significantly influence biomass recalcitrance and sugar release.

Although studies have shown that low lignin content generally increases the ability of cellulolytic enzymes to hydrolyze plant biomass [13,40,41] and considerable efforts have been taken to reduce lignin content through genetic engineering, some have also found there is no clear trend between lignin content in plant biomass and its recalcitrance to sugar release. For example, Voelker et al. reported no substantial changes in saccharification potential of field-grown hybrid poplar with low lignin levels due to downregulation of 4CL gene in lignin biosynthesis [42]. On the other hand, Chen and Dixon [43] reported that genetically engineered alfalfa lines with lower lignin content and modified lignin structures [44] demonstrated improved fermentable sugar yields when compared to wild-type plants, although the digestibility of these transgenic lines were also shown to be similar despite differences in lignin content of mutants. Shen et al. [45] reported that ectopic overexpression of PvMYB4 genes in switchgrass resulted in reduced lignin content and ester-linked *p*-coumarate:ferulate ratio in lignin and an approximately threefold increase in sugar release efficiency from transgenic cell wall residues. While high S/G ratios are usually considered favorable for deconstruction of angiosperms [46], the reverse is true for genetically engineering alfalfa, tall fescue and switchgrass [43,47,48]. In some cases, lignin content appears to be more responsible for recalcitrance than lignin composition while at times the opposite is true [43]. In addition, research efforts have also been focusing on the biosynthesis and incorporating alterative phenolic monomers into lignin through genetic engineering to alter the structure of lignin polymer to facilitate lignin removal from lignocellulosic biomass by pretreatments or to improve the penetration and action of hydrolytic enzyme [49,50]. Vanholme et al. [49] suggested that it would be desirable that bioenergy crops contain genetically engineered/tailored lignin that is readily degraded by pretreatments but that this mutant lignin could still fulfill its biological role in plants. A recent study by Elumalai et al. [51] reported that epigallocatechin gallate (EGCG) was readily copolymerized with monolignols to become integrally cross-coupled into cell wall lignin where it greatly enhanced alkaline delignification and subsequent enzymatic saccharification. Eudes et al. [52] describes a new strategy developed in Arabidopsis to enhance the biosynthesis and incorporation of side-chain-truncated lignin monomers as DP reducers into lignin polymers to reduce lignin polymerization and decrease cell wall recalcitrance to enzymatic hydrolysis. These results further demonstrate that multiple factors can considerably influence biomass recalcitrance to sugar release from wild type and genetically engineered transgenics. Thus further studies are needed to understand and optimize these competing effects. The observed differences in the impact of native vs. transgenic lignin with respect to recalcitrance need to be further investigated as their molecular mechanisms are yet to be fully defined.

## Hemicelluloses hydrolysis and pectins

During dilute acid and hydrothermal pretreatment, the hydronium ions released by the acid or water cause depolymerization of hemicellulose by selective hydrolysis of glycosidic linkages, liberating *O*-acetyl group and other acid moieties to form acetic and uronic acids. The release of these acids is thought to catalyze the hydrolysis of hemicelluloses and oligosaccharides, particularly in hydrothermal pretreatment [53,54]. Xylan, the main hemicellulose in hardwoods and annual plants, is hydrolyzed to xylose or xylo-oligomers during DA or hydrothermal pretreatment respectively, whereas glucomannan is relatively stable in acidic process (Table 1) [18,55-57]. In general, the degree of xylan hydrolysis increases as the DA or hydrothermal pretreatment severity increases, as shown in Table 1[58-60]. Xylan is dissolved in the reaction media first as high molecular weight (DP > 25) material followed by cleavage of more bonds between xylose residues upon higher pretreatment severity. The initially dissolved high DP xylo-oligomers have a high degree of acetylation since the acetyl groups increase xylan solubility [61]. The medium molecular weight (DP 9-25) xylo-oligomers are predominate in hydrothermal pretreatment, and their proportions decrease slightly as severity increases due to increased decomposition [53,54]. On the other hand, most of the released xylan is accumulated in the reaction medium in the form of xylose during lower severity conditions for DA pretreatment. The more severe the pretreatment, the more low molecular weight (DP < 9) xylo-oligomers relative to high molecular weight xylo-oligomers are detected in the reaction media for both DA and hydrothermal pretreatment. However, increasing DA and hydrothermal pretreatment severity also increases the risk of xylan degradation to furfural, which is a by-product inhibitory to the formation of ethanol during fermentation. In addition, degradation of hemicellulose during DA pretreatment can contribute to the formation of pseudo-lignin, which is even more detrimental to enzymatic hydrolysis than pretreated lignin [25,26,62].

**Table 1:** **Residual hemicellulose content versus pretreatment condition for different substrates**[57,59]

**Feedstock**	**Temperature (°C)**	**Residence time (min)**	**H**_**2**_**SO**_**4**_**(% w/w)**	**Residual hemicellulose (%)**
Wheat straw	170	15	2.5	8
	160	15	2.5	20
	190	10	1.0	43
	180	2	0	93
	190	30	0	52
	200	20	0	35
Loblolly pine	150	60	0	90
	150	60	1.0	40

It has been reported that conducting DA or hydrothermal pretreatment in a flow-through configuration removes hemicellulose and lignin and produces a more digestible substrate than the conventional batch reactor system [16,63-66]. Although the detailed mechanisms that control hemicellulose removal during hydrothermal pretreatment are not well understood, the mass transfer effect controlled by the flow-rate, lignin-hemicellulose-oligomers and their solubility are believed to be significant. It was observed that the distribution of solubilized xylan shifted toward higher DP oligomers as the hydrothermal pretreatment operation changed from batch to low, and then high flow-rate of water, as shown in Table 2[65]. This is attributed to the greater amount of water dissolving larger oligomers at higher flow rates and the rapid removal of dissolved oligomers from the reactor by the higher flow rate before the oligomers can hydrolyze further [65]. This leads to higher overall xylan recovery from flow-through reactor compared to batch configuration, because some in-situ xylose degradation occurs in batch reactor due to longer exposure to high temperatures. Despite the advantages of flow-through configuration, high water and energy consumption and the difficulty in equipment development might impede commercial applications of this method. As a result, further studies are needed to understand DA and hydrothermal pretreatment from a fundamental scientific prospective, in order to optimize pretreatment conditions.

**Table 2:** **Yield of xylan oligomers (DP < 30) and total xylan recovery in the hydrolysate after treatment of corn stover at 200°C for 10 min**[65]

	**Yield (%)**			
**Flow rate (ml/min)**	**Total xylan recovery**	**DP 1-30**	**DP > 30**	**Ratio of short chain to long chain oligomers**
0 (batch)	38.1	28.1	10.0	2.8
2	48.2	20.3	27.9	0.7
25	73.3	9.1	64.2	0.1

Hemicellulose has been considered to contribute to biomass recalcitrance by covering and protecting the cellulose fibrils from enzymatic deconstruction. In addition, xylans have high affinity to cellulose and can absorb irreversibly on cellulose surface [67]. Several studies have indicated that removing a high percentage of hemicellulose can increase the enzymatic digestibility of cellulose [58,68]. DA and hydrothermal pretreatment hydrolyzes hemicellulose, increasing the accessibility of cellulose to cellulases and consequently increasing the degree of enzymatic hydrolysis of cellulose. Recent studies also showed that xylo-oligomers inhibit cellulase action and have stronger inhibition effects on the initial rate of enzymatic hydrolysis of cellulose than xylan or xylose at similar concentrations and that cellulases bind more strongly to xylan than cellulose [69-71]. As a result, xylan and xylo-oligomers appear to reduce cellulase reactivity towards cellulose by undesirable association with cellulases. Therefore, the removal of hemicellulose during DA and hydrothermal pretreatment prior to enzymatic hydrolysis not only increases cellulase accessibility but also reduces cellulase inhibition by xylo-oligomers during enzymatic hydrolysis, contributing to the reduction of recalcitrance in the pretreated biomass.

Hemicellulose chains are typically extensively acetylated, and acetyl groups have been shown to increase lignocellulosic recalcitrance [72]. Pan et al. [73] suggested that acetyl groups inhibited productive bindings of cellulases to cellulose by restricting cellulase accessibility to cellulose. Selig and coworkers [74] showed that acetyl groups bound to the xylan backbone hindered cellulase access to the β-1,4 glycosidic linkages. During DA and hydrothermal pretreatment, the behavior of acetyl release to the reaction medium versus the pretreatment severity is similar as that of xylan release [55,75]. It was reported that the hydrolyzed acetyl groups became an in-situ source of acetic acids that further catalyzes xylan depolymerization, whereas another fraction of the acetyl esters remained covalently linked to the xylan backbone and were released from the residue together with the xylan as esterified xylo-oligomers [66]. The additional acetyl groups from dissolved xylo-oligomers can be cleaved at higher pretreatment severity conditions such as longer residence time. Deacetylation by DA and hydrothermal pretreatment is favorable because deacetylation not only provides more sites for enzyme attack, but also reduces recalcitrance through the formation of more easily hydrolyzed xylo-oligomers with few side branches, thereby increasing xylose yield and consequently improving enzymatic digestibility of pretreated biomass [72,76].

Recently, DeMartini et al. [21] employed a novel glycome profiling technique in which cell wall glycan-directed monoclonal antibodies were applied to monitor deconstruction and structural changes involving major classes of polysaccharides in *Populus* biomass during hydrothermal pretreatment of different lengths at 180°C. Glycome profiling results demonstrate that hydrothermal pretreatment causes an initial significant loss of pectic and arabinogalactan epitopes in concert with disruption of lignin-polysaccharide interactions, namely lignin-pectin/arabinogalactan interactions, followed by significant removal of xylans and xyloglucans at longer pretreatment times. The initial disruption of lignin-arabinogalactan/pectin in concert with other changes ( some lignin-xylan interactions disruptions and the loss of arabinogalactans) that occurred in the cell wall were associated with an increase in digestibility of up to 24% as compared to the untreated material, depending on enzyme loading.

## Cellulose structural alterations

Cellulose is a linear polymer made up of β-D-glucopyranosyl units linked with 1 → 4 glycosidic bonds with cellobiose as the repeating unit. Each D-anhydroglucopyranose unit possesses hydroxyl groups at C2, C3, and C6 positions. Cellulose has a strong tendency to form intra- and inter-molecular hydrogen bonds between the molecules of cellulose. The hydrogen bonds in the linear cellulose chains promote aggregation into a crystalline structure and give cellulose a multitude of partially crystalline fiber structures and morphologies [77,78]. The ultrastructure of native cellulose (cellulose I) has been discovered to possess complexity in the form of two crystal phases: I_α_ and I_β_[79]. In addition to the crystalline and amorphous regions, native cellulose is also proposed to contain a para-crystalline portion which has more order and less mobility than amorphous chain segments but is less ordered and more mobile than the crystalline domain [80,81]. Cellulose crystallinity and DP have been considered major biomass recalcitrance features that affect enzymatic hydrolysis performance.

### Cellulose crystallinity

During dilute acid and hydrothermal pretreatment, the hydrolyzation of cellulose and subsequent solubilization of glucose can result in an increase of cellulose crystallinity index (CrI) in biomass, as shown in Table 3. Foston et al. [82] have observed the para-crystalline content of cellulose in poplar and switchgrass appears to increase during the DA pretreatment. They suggested that the majority of the increase in crystallinity and para-crystalline percentage is primarily due to localized hydrolyzation and removal of cellulose from the amorphous regions. It has been proposed that cellulose I_α_ is primarily converted to para-crystalline cellulose during DA pretreatment, followed by conversion of para-crystalline cellulose to cellulose I_β_[82]. Similarly, Sannigrahi et al. [22] compared the crystalline index of Loblolly pine cellulose before and after two-stage DA pretreatment and observed a large increase in the relative proportion of cellulose I_β_ accompanied by a decrease in the relative proportions of both cellulose I_α_ and para-crystalline region. Likewise, Cao et al. [24] reported that the crystalline index of poplar cellulose remained almost unchanged during the early DA pretreatment of short time (0.3 - 5.4 min); as the pretreatment time extended to 8.5 min, the cellulose had a slight increase of crystalline index (increase by ~ 3 units). It should be noted that the crystallinity increase reported by Cao et al. [24] was smaller than those reported by Foston and Sannigrahi after DA pretreatment. This might be due to the reason that the DA pretreatment conditions applied by Foston and Sannigrahi were at higher severity than those of Cao et al. In addition, Yu et al. [83] have found the hydrothermal pretreatment temperature has significant impact on the amorphous and crystalline cellulose degradation. They observed that the minimal temperature required to rupture the glycosidic bonds in the chain segments within the amorphous portion of cellulose appeared to be approximately 150°C, whereas for the crystalline portion of cellulose it was 180°C. This difference in the hydrolysis behavior between amorphous and crystalline cellulose was attributed to the ultrastructural differences in the amorphous and crystalline portions of cellulose.

**Table 3:** Cellulose crystallinity index (CrI) before and after DA and hydrothermal pretreatments for different substrates

**Substrate**	**Pretreatment conditions**	**CrI (%) before pretreatment**	**CrI (%) after pretreatment**	**Reference**
Rice straw	DA pretreatment: 1% H_2_SO_4_, 180°C, 4 min	57.0^a^	65.0^a^	[84]
Poplar	DA pretreatment: 2% H_2_SO_4_, 190°C, 70 s	49.9^a^	50.6^a^	[32]
Corn stover	DA pretreatment: 3% H_2_SO_4_, 180°C, 90 s	50.3^a^	52.5^a^	[32]
Loblolly pine	DA pretreatment: 1^st^ stage: 0.5% H_2_SO_4_, 180°C, 10 min; 2^nd^ stage: 1.0% H_2_SO_4_, 200°C, 2 min.	62.5^b^	69.9^b^	[22]
Switchgrass	DA pretreatment: 5% H_2_SO_4_, 190°C, 1 min	44.0^b^	52.0^b^	[85]
Poplar	Hydrothermal pretreatment: 200°C, 10 min	49.9^a^	54.0^a^	[32]
*Tamarix ramosissima*	Hydrothermal pretreatment: 180°C, 9 min	41.0^a^	51.4^a^	[86]
Costal Bermuda grass	Hydrothermal pretreatment: 170°C, 60 min	50.2^a^	69.4^a^	[87]

a: CrI was measured by X-ray diffraction (XRD) method [88].

b: CrI was measured by solid-state NMR technique [89].

It is generally accepted that amorphous cellulose presents less resistance to enzyme depolymerization in the cellulose-to-glucose conversion than crystalline cellulose. However, the interpretation of data published in the literature on cellulose enzymatic hydrolysis in terms of CrI is not straightforward in terms of providing a clear indication of the digestibility of a biomass sample. Chundawat et al. [90] have compared the effects of several leading chemical pretreatments that result in enhanced cell wall digestibility. The data demonstrates that while DA, hydrothermal, steam explosion and lime pretreatments generally result in relative increase in cellulose crystallinity with respect to untreated control, ammonia recycle percolation (ARP) and ammonia fiber expansion (AFEX) pretreatments show a relative decrease in cellulose crystallinity after pretreatment. Recently, Park et al. [89] investigated the impact of crystallinity on the cellulose digestibility during the enzymatic hydrolysis of pretreated biomass. They suggest that there is no clear correlation between the CrI and cellulose digestibility since the cellulose accessibility is not only affected by cellulose crystallinity but also by several other parameters, such as lignin/hemicellulose contents and distribution, porosity, and particle size. In addition, the crystallinity was usually coupled with changes to other biomass properties and differences in observed enzyme hydrolysis kinetics after thermal-chemical pretreatment may be governed by the combined effects. Consequently, CrI alone may not adequately explain differences in observed hydrolysis rates and should be considered just one of several parameters that likely affect the enzymatic hydrolysis rate of cellulose in a biomass sample. Nonetheless, the role of cellulose crystallinity and its relationship to acidic pretreatments must now be revised. For the long time, it was envisaged that DA and autohydrolysis pretreatments were successful in reducing recalcitrance, in part, by significantly lowering the crystallinity of cellulose and this effect, we now know, is incorrect.

### Cellulose degree of polymerization

DA and hydrothermal pretreatments result in partial hydrolyzation of cellulose leading to a reduction of DP especially at high-severity pretreatment conditions, which increases the enzymatic digestibility of cellulose, as shown in Table 4. The DP of cellulose from different substrates usually decreases gradually until reaching a nominal value, namely, the leveling-off degree of polymerization (LODP) throughout the course of pretreatment [91-94]. The initial DP reduction period is believed to represent the hydrolysis of the reactive amorphous region of cellulose, whereas the slow plateau rate phase corresponds to the hydrolysis of the slowly reacting crystalline fraction of cellulose [91]. Cao et al. [24] observed a reduction in molecular weight of cellulose during DA pretreatment of poplar at 170°C with ~ 86% reduction of DP reached at around 27 min. In addition, the pretreatment severity has significant influence on the DP decrease. For example, recent research indicated the low-DP glucose oligomers are produced at 180°C during hydrothermal pretreatment, whereas large-DP glucose oligomers are released at temperatures above 200°C [83].

**Table 4:** Cellulose DP before and after DA and hydrothermal pretreatments for different substrates

**Substrate**	**Pretreatment conditions**	**DP before pretreatment**	**DP after pretreatment**	**Reference**
Corn stover	DA pretreatment: 3% H_2_SO_4_, 180°C, 90 s	7300^a^	2700^a^	[32]
Poplar	DA pretreatment: 2% H_2_SO_4_, 190°C, 70 s	3500^a^	600^a^	[32]
Loblolly pine	DA pretreatment: 1% H_2_SO_4_, 180°C, 30 min	3642^b^	1326^b^	[95]
Switchgrass	DA pretreatment: 5% H_2_SO_4_, 180°C, 5 min	1891^b^	1342^b^	[82]
Corn stover	Hydrothermal pretreatment: 190°C, 15 min	7300^a^	5700^a^	[32]
Poplar	Hydrothermal pretreatment: 200°C, 10 min	3500^a^	1750^a^	[32]

a: DP was measured by viscometric method [96].

b: DP was measured by gel-permeation chromatography (GPC) technique [97].

Lower DP was observed to improve cellulose digestibility during enzymatic hydrolysis mainly due to the increase of cellulose chain reducing ends [98]. As the DP of cellulose decreases, the number of reducing ends of cellulose increases, thus allowing for more exoglucanase effective activity. For example, Martínez et al. found that the enzymatic saccharification increased with reduction in cellulose DP [99]. Furthermore, shorter chains make cellulose to be more amenable to enzymatic deconstruction because they do not form strong hydrogen bonding network (i.e., they form weaker networks permitting greater possibility for enzyme access) [100,101]. Thus, decreasing cellulose DP during DA and hydrothermal pretreatments reduces the biomass recalcitrance and favors the cellulose-to-glucose bio-conversion.

## Biomass porosity

Cellulose accessibility to cellulases is also largely limited by the anatomical structure of plant cell wall. Specifically, it is the pores existing in the plant cell walls that allow cellulases to access the surface of cellulose microfibrils. The specific surface area and the mean pore size are influential structural features related to cellulase adsorption on the cellulose surface and subsequent enzymatic deconstruction [102,103]. It was reported that pore size larger than 3 nm had an essential accessibility effect for cellulase protein molecule into the plant cell wall [104]. Several studies have indicated that the breakdown and loosening of the lignocellulosic structure by DA and/or hydrothermal pretreatments increase the specific surface area, pore volume and pore size of the biomass (Table 5) [105-110]. Hsu et al. [106] suggested that this was not only caused by hemicellulose removal but also by hydrolysis and rearrangement of the lignin structure. A further study by Foston and Ragauskas [107] revealed that the increase in pore size during DA pretreatment was due to existing pores within the system expanding rather than generating new pores. Chen et al. also investigated the impact of dilute sulfuric acid pretreatment on particle size of sugarcane bagasse and observed a decrease in average particle size and an increase in specific surface area of the biomass under the environment of microwave irradiation for 5 min [108]. The authors suggested that the lignocellulosic structure of biomass simultaneously underwent fragmentation and swelling during pretreatment with fragmentation releasing small components, thereby enlarging the specific surface area. However, with the pretreatment time extending to 10 min the swelling behavior of biomass became more drastic, resulting in a lower specific surface area than that at 5 min (Table 5). These results further suggest optimization of DA pretreatment conditions is essential to open the plant cell wall structure and expose cellulose fibrils, in order to increase enzymatic digestibility of pretreated biomass.

**Table 5:** **Specific surface area and pore volume before and after pretreatment for different substrates**[108,109]

**Substrate**	**Pretreatment conditions**	**Specific surface area (m**^**2**^**/g)**	**Pore volume (ml/g)**
Rice straw	untreated	1.33	0.004
	130°C, 2% H_2_SO_4_, 15 min	4.48	0.012
	150°C, 2% H_2_SO_4_, 4 min	5.35	0.020
	160°C, 2% H_2_SO_4_, 2 min	5.76	0.022
	170°C, 2% H_2_SO_4_, 1 min	8.94	0.027
Sugarcane bagasse	untreated	1.00	NA
	130°C, 2% H_2_SO_4_, 5 min	1.80	NA
	160°C, 2% H_2_SO_4_, 5 min	2.38	NA
	190°C, 2% H_2_SO_4_, 5 min	6.31	NA
	160°C, 2% H_2_SO_4_, 10 min	0.98	NA
	190°C, 2% H_2_SO_4_, 10 min	5.00	NA

## Summary and conclusions

In summary, dilute acid and hydrothermal pretreatments lead to substantial structural changes of lignin, hemicellulose and cellulose in lignocellulosic biomass. Lignin removal, β-O-4 cleavage, shift of S/G ratio, hemicellulose removal, changes in cellulose DP and crystallinity, as well as porosity are among the most significant structural alterations observed in pretreated biomass. Given the rigid and complex spatial cell wall structure constructed by intimate linking of its chemical compositions, interactive effects naturally exist between these factors and altering one structural feature is accompanied by change of additional ones during dilute acid and hydrothermal pretreatments. It appears that there is no signal, independent chemical or structural factor that exclusively controls biomass recalcitrance. This observation may well be due to the fact that biomass accessibility to deconstruction enzymes is a key controlling factor which in turn can be influenced by the chemical compositional components described above. This issue needs to be further explored and defined in the upcoming years to provide a firm foundation by which pretreatment and biological deconstruction can be rationally optimized from first principles.

## Competing interests

The authors declare that they have no competing interests.

## Authors’ contributions

This review was written in a collaborative, team manner, hence it was jointly prepared by YP, FH, FH, BHD, and AJR. All authors read and approved the final manuscript.
